# Acetonæmia and Acetonuria

**Published:** 1907-11-23

**Authors:** 


### Points in Pathology.

### ACETONÆMIA AND ACETONURIA.

The fact that acetone can occur in the urine in a great many other conditions besides glycosuria and diabetes is far from being well known; and yet acetonuria, whenever it occurs, must always be of clinical importance.

We do not propose to give more than the gist of all the work that has been done upon the subject; the four most important points which have been established are the following:—

1. A healthy man on ordinary mixed diet passes no clinically recognisable quantity of acetone in his urine.

2. A healthy man, if kept without food, or if given a diet containing absolutely no carbohydrate at all, begins to pass acetone in his urine almost as soon as the products of the last carbohydrate meal have been got rid of.

3. Acetone is only one of many allied products of abnormal metabolism; two other products of a similar nature being oxybutyric acid and diacetic acid. The accumulation of these substances in the body may give rise to symptoms which have been collectively termed "acidosis."

4. The source of the acetone and its allies is fat, and not carbohydrate nor proteid; at any rate, fat is their main source; and acidosis may occur whenever there is any condition when the patient is compelled to metabolise his fat to an excessive extent.

If a healthy man who voluntarily starves himself begins to pass acetone in his urine, it is little wonder that many patients who are involuntarily starved owing to the nature of their diseases do likewise. Children suffering from severe diarrhœa and vomiting; patients who vomit continually from anæmia, from gastric or duodenal ulcer, from pregnancy, and so forth, will often be found to have acetonuria. It does not follow, of course, that the amount of acetone and allied substances produced in these cases are sufficient to cause actual symptoms of acidosis. The symptoms of the latter may be latent, but they may be added to those of the other troubles.

We need not enumerate all the conditions in which acetonuria may be found; we have indicated how much more common it is than might be expected; all we need is an easy test for its detection.

The oldest test recommended for the detection of acetone was the addition of iodine in an aqueous solution of potassium iodide, when on warming *iodoform* should be formed and should be recognised by its smell. This test, however, is only applicable as a rule when the clinician has an apparatus for distilling the urine; the acetone is very volatile, so that it comes over with the first few c.c. of water, and the iodoform test can then be applied with some ease to the concentrated solution of acetone so obtained. If the iodoform test is applied to urine without previous distillation it will seldom come off.

A simpler and more practical test is that with sodium nitroprusside and caustic soda. If two test-tubes are taken, and into the first is put some perfectly normal urine, and into the second some urine known to contain acetone, and then to both about ten drops of caustic soda solution, followed by 20 drops of strong solution of sodium nitro-prusside, are added, each will go a bright reddish-brown colour owing to the presence of creatinin; if strong acetic acid be now added drop by drop, the red colour due to the creatinin in the normal urine will entirely disappear, whereas the red colour in the tube containing acetone will deepen into a dark claret-red colour—so dark as to be almost impervious to light in some cases. It is this deepening of the red colour which is the easiest test for acetone at present known; it is a very pretty test-tube reaction.

One important group of conditions in which acidosis is as yet little recognised, though it is of great importance, is in connection with anæsthetics. The kind of anæsthetic used matters little, but the length of time during which anæsthesia is maintained matters much; hence with gas the acidosis is negligible, while with ether, chloroform, and the A.C.E. mixture it is very far from being so. For some hours after a long anæsthetic, acetone and its allies may be found in the stomach contents and in the urine; and recent work goes far to show that many of the bad symptoms that result from anæsthetics are due, in part at least, to this acidosis. The condition known as delayed chloroform poisoning is attributed to it; and there can be no doubt that the excessive and persistent vomiting that may follow anæsthetics is partly due to the acetonæmia, and to the presence of acetone in the stomach. This well explains the wonderful relief that is to be obtained by simple lavage of the stomach in the worst cases; and seeing that the trouble is due to poisoning by organic acids, it is not surprising that the benefit of giving the patient a dilute solution of carbonate of soda to drink, or of using such a solution for the gastric lavage, is greater than that obtained when plain water is employed. Patients' lives have again and again been saved by the simple use of sodium carbonate in weak solution in this way.

We have not space to do more than indicate the kind of ways in which an examination of the urine for acetone may prove helpful in the treatment of different conditions. There is one further point, however, that we would lay stress upon here, and that is the great danger there is of suddenly cutting off the carbohydrates from the dietary of a patient with diabetes. Diabetic coma is directly due to poisoning by acetone or its allies. A diabetic patient is always liable to pass acetone; a healthy man is certain to pass acetone if he is starved of carbohydrates; how much more certainly a patient who has diabetes is to form increased, and possibly fatal, quantities of acetone and similar toxic products of metabolism if his carbohydrates are completely cut off! The amount of sugar in a diabetic urine is almost unimportant provided no acetone is present. It is much better that the patient should pass much sugar and no acetone, than that his sugar should be decreased at the expense of acetone formation and possibly fatal acidosis.

